# Engineering Artificial Somatosensation Through Cortical Stimulation in Humans

**DOI:** 10.3389/fnsys.2018.00024

**Published:** 2018-06-04

**Authors:** Brian Lee, Daniel Kramer, Michelle Armenta Salas, Spencer Kellis, David Brown, Tatyana Dobreva, Christian Klaes, Christi Heck, Charles Liu, Richard A. Andersen

**Affiliations:** ^1^Department of Neurological Surgery, Keck School of Medicine of USC, University of Southern California, Los Angeles, CA, United States; ^2^USC Neurorestoration Center, Keck School of Medicine of USC, Los Angeles, CA, United States; ^3^Department of Biology and Biological Engineering, California Institute of Technology, Pasadena, CA, United States; ^4^Department of Neurology, Keck School of Medicine of USC, University of Southern California, Los Angeles, CA, United States; ^5^Tianqiao and Chrissy Chen Brain-Machine Interface Center, Chen Institute for Neuroscience, California Institute of Technology, Pasadena, CA, United States

**Keywords:** somatosensation, cortical stimulation, brain machine interface (BMI), sensory feedback control, electrocorticography (ECoG)

## Abstract

Sensory feedback is a critical aspect of motor control rehabilitation following paralysis or amputation. Current human studies have demonstrated the ability to deliver some of this sensory information via brain-machine interfaces, although further testing is needed to understand the stimulation parameters effect on sensation. Here, we report a systematic evaluation of somatosensory restoration in humans, using cortical stimulation with subdural mini-electrocorticography (mini-ECoG) grids. Nine epilepsy patients undergoing implantation of cortical electrodes for seizure localization were also implanted with a subdural 64-channel mini-ECoG grid over the hand area of the primary somatosensory cortex (S1). We mapped the somatotopic location and size of receptive fields evoked by stimulation of individual channels of the mini-ECoG grid. We determined the effects on perception by varying stimulus parameters of pulse width, current amplitude, and frequency. Finally, a target localization task was used to demonstrate the use of artificial sensation in a behavioral task. We found a replicable somatotopic representation of the hand on the mini-ECoG grid across most subjects during electrical stimulation. The stimulus-evoked sensations were usually of artificial quality, but in some cases were more natural and of a cutaneous or proprioceptive nature. Increases in pulse width, current strength and frequency generally produced similar quality sensations at the same somatotopic location, but with a perception of increased intensity. The subjects produced near perfect performance when using the evoked sensory information in target acquisition tasks. These findings indicate that electrical stimulation of somatosensory cortex through mini-ECoG grids has considerable potential for restoring useful sensation to patients with paralysis and amputation.

## Introduction

The incidence of new cases of spinal cord injury in the United States is estimated to be 12,000 per year. When considered with strokes, neuropathies and limb amputations, the prevalence of loss of limb function, especially in the upper extremities, is extensive. To restore function to such individuals, brain-machine interfaces (BMIs) are being designed to extract motor execution signals from cortex and decode them to operate physical or virtual effectors (Andersen et al., 2004a,b, 2010; Hochberg et al., 2006; Andersen and Cui, 2009; Kim et al., 2011; O’Doherty et al., 2011; Simeral et al., 2011; Lee et al., 2013). Animal and human research over almost four decades has made the possibility of controlling external devices with neural activity a reality.

**Figure 1:**
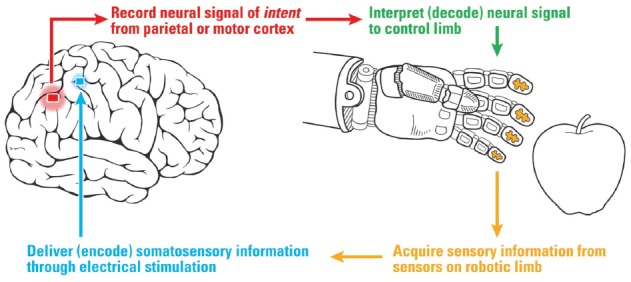
Diagram of a cortical-based brain-machine interface for neuroprosthetics with sensory feedback to the somatosensory hand area. Sensors on the robotic hand provide information via electrical stimulation of the hand area of somatosensory cortex (S1).

In recent years, there has been substantial interest in providing somatosensory feedback to create a “closed-loop” system for BMIs using artificially generated somatosensory feedback. For example, a bidirectional BMI to operate a robotic hand could read out signals from touch sensors in the hand, then use stimulation of primary somatosensory cortex (S1) to write this information directly into the brain (Figure 1). Ideally, by stimulating the brain’s existing somatosensory processing area, these signals would be more naturally integrated into the motor-sensory control process, leading to enhanced performance and an improved sense of embodiment. As cognitive-based motor neuroprosthetics have garnered attention (Klaes et al., 2014), and touch sensors are available in commercial robotic limbs (Wettels et al., 2008), integrating artificial sensation into a cognitive neural prosthesis has become a realizable possibility (Fagg et al., 2009; Marzullo et al., 2010; O’Doherty et al., 2012). Ideally, the external prosthesis would become incorporated into the patient’s body schema, featuring both motor control and somatosensory feedback (Gallagher and Cole, 1995; Botvinick and Cohen, 1998; Tsakiris and Haggard, 2005; Arzy et al., 2006).

Some success has been achieved in generating artificial sensation with nonhuman primates (NHPs). NHPs trained in active exploration tasks have been able to use artificial stimulation to discriminate between periodic pulse trains of intracortical microstimulation (ICMS; O’Doherty et al., 2012). Additionally, varying the frequency of ICMS to replace physical stimuli, NHPs performed a vibrational “flutter” discrimination task with nearly the same degree of accuracy (Romo et al., 1998, 2000). Work in NHPs by Kim et al. (2015a,b) has been instrumental to determine the effect of stimulus parameters and electrode configuration on detection thresholds and discrimination levels. Integration of such artificial sensation has been accomplished recently in true closed-loop BMIs (O’Doherty et al., 2011; Klaes et al., 2014). Operating a virtual effector, NHPs were able to successfully discriminate between targets using only artificial “textural” clues from cortical stimulation (O’Doherty et al., 2011), and use the information conveyed in the ICMS pulses to perform variations of center-out tasks (Dadarlat et al., 2015).

Translation of these NHP studies to human patients is still underway. Use of ICMS is limited by its invasiveness and the potential damage of the cortex. Another significant limitation is that implanted micro-electrode arrays physically span a small extent of the somatotopic representation of the hand in human cortex. The use of less invasive electrocorticography (ECoG) grids that cover a larger area of cortex may mitigate some of these limitations. For example, a recent study by Hiremath et al. (2017) showed the use of a high-density ECoG grid to evoke somatosensory percepts in a paralyzed subject. They tested the effects of stimulation parameters in these sensory percepts, and hinted how different parameters might affect the type of sensation elicited. However, the replicability of the sensations and the stability of the parameters’ effects in other subjects must be confirmed. With slightly smaller dimensions, micro-ECoG grids could offer both higher spatial density and good cortical coverage (Lycke et al., 2014), but additional materials and device research will be required to address the risk of high charge densities on such small electrodes (Pazzini et al., 2017). Other studies have used standard ECoG grids to demonstrate subjects’ abilities to discriminate different frequencies (Johnson et al., 2013) and use artificial sensations during a simple BMI task (Cronin et al., 2016). However, these experiments involved two and one subject, respectively, and did not target specific hand or arm receptive fields.

This study describes electrical stimulation over somatosensory cortex with high-density mini-ECoG grids in nine patients monitored for epilepsy. A primary goal of the study is to determine whether stimulation through mini-ECoG grids using clinically-approved parameters could produce robust artificial sensations across the patient population (Ray et al., 1999). The study specifically targeted percepts in the hand area, to test whether these fields follow expected hand somatotopy and to evaluate whether the intensity of perception could be altered by manipulating the stimulus parameters. To demonstrate the potential for integrating this new information into behavioral routines, subjects were additionally asked to use the somesthetic percepts in a target localization task.

## Materials and Methods

### Subjects

Nine epilepsy patients (2 males, ages 21–62, mean 38 years old) undergoing Phase II ECoG monitoring for seizure localization participated in the study (Table 1). This study was carried out in accordance with the recommendations of University of Southern California Health Sciences Campus Institutional Review Board with written informed consent from all subjects. All subjects gave written informed consent in accordance with the Declaration of Helsinki. The protocol was approved by the University of Southern California Health Sciences Campus Institutional Review Board.

**Table 1:** Subject demographics.

Subject ID	Implant hemisphere
01	Right
02	Left
03	Left
04	Right
05	Right
06	Left
07	Right
08	Left
09	Right

*Summary of subjects participating in this study*.

**Figure 2:**
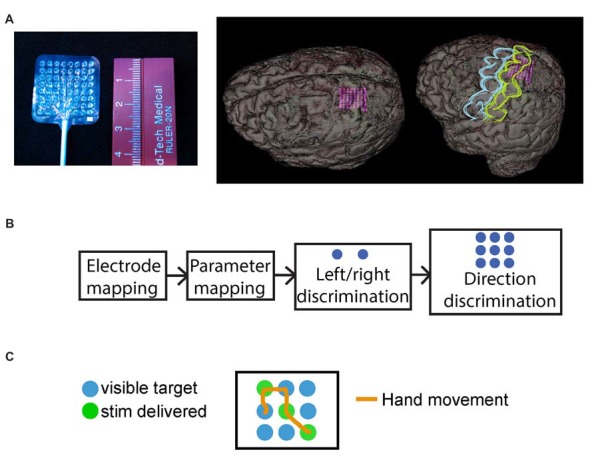
Mini-electrocorticography (mini-ECoG) grid and experimental paradigm. **(A)** On the left, the Ad-Tech “mini” electrocorticography grid used, with 64 2-mm platinum contacts, spaced 3 mm center-to-center. On the right, reconstructed images of the grid placement for S05, with an oblique and an overlaid representation of where primary motor cortex (blue) and primary somatosensory cortex (yellow) are in relation to the mini-grid. **(B)** Schematic of typical session timeline, from initial electrode mapping to behavioral tasks. **(C)** Exemplar trial for the “directional” behavioral task with 3-by-3 grid, where the green circles illustrate the underlying direction followed by the epileptologist to trigger stimulation, these were not visible to the subjects. The orange line shows a mock hand trajectory of subjects hand over the grid which started at one of the non-stimulated (blue), locations.

All patients underwent a standard craniotomy in the frontotemporoparietal region and placement of subdural electrode grids and strips for seizure mapping. In all instances, the somatosensory cortex, including the hand area, was accessible from the craniotomy. Data for this study were collected from high-density mini-ECoG grids (64-contact, 8 × 8 grid, 2-mm contact with 3-mm spacing; Adtech FG64C-MP03) placed on the somatosensory hand area, based upon preoperative MRI localization with intraoperative frameless navigation (see Figure 2A). This MRI-compatible grid is FDA-approved for recording and stimulation in humans. The dura was closed over the electrode grid and the cranium replaced. The leads of the electrodes were tunneled through the scalp and sutured to the skin to hold them in place. The scalp was sutured closed and the incision dressed. Figure 2A shows the reconstructed image of the grid placement for Subject 5.

After implantation, the subjects were then transferred to the intensive care unit (ICU) and monitored for seizure activity. In the ICU, the seizure foci were mapped and various parts of the clinical standard ECoG grid were stimulated and mapped to determine both the resection target and critical structures to be avoided during resection. Both the mini and clinical ECoG grids were connected to a clinical electroencephalography (EEG) machine. Cortical stimulation was performed manually by the epileptologist using a Grass Technologies S12X Cortical Stimulator (Natus Neurology Incorporated, Warwick, RI, USA). After approximately 7 days in the epilepsy monitoring unit, the patients returned to the operating room for grid removal and for seizure focus resection if clinically appropriate.

Figure 2B illustrates a typical session timeline for testing our subjects. First, after initial consultation with an epileptologist, we selected a grid region most likely to reside over the hand area, and proceeded to map the mini-ECoG grid. Next, we selected a single pair of electrodes for stimulus parameter mapping using bipolar stimulation. Finally, using the same electrode pair and the lowest stimulation current that elicited a reliable sensation, the subjects performed the behavioral tasks.

### Cortical Stimulation: Grid Mapping

Cortical stimulation trials took place during ICU monitoring. The mini-ECoG grid was mapped to find reliable sensory receptive fields, and find the boundaries between primary motor and somatosensory cortices. Various stimulation parameter combinations were tested to find the optimal settings for patient response and comfort (Table 2). These parameters were carefully selected in accordance with manufacturer and clinical recommendations and the safety limits established in the IRB-approved protocol, and fell within widely accepted safety ranges established in the epilepsy literature (Agnew and McCreery, 1987; Wyllie et al., 1988; Ray et al., 1999; Signorelli et al., 2004). During this initial grid mapping, parameters varied at the discretion of the epileptologist and did not reach the maximum approved values (amplitude range: 2–6 mA; duration range: 0.5–2 s). For each patient, we identified clear sensory hand areas, and identified different receptive fields across most digits and the palm. We selected the electrode pair whose stimulation produced the most consistent receptive fields and used this pair of electrodes for the remainder of the experiment. If multiple reliable receptive fields were found, then the one closest to the ventral surface of the tips of the digits was used (Table 3).

**Table 2:** The range of pulse parameters used for mapping and testing.

Stimulation parameters	Parameter range
Polarity	positive, negative, alternating
Pulse width	100 μs to 2000 μs (300 μs)
Current	1 mA to 10 mA (1 mA)
Frequency	2 Hz to 100 Hz (20 Hz)
Duration	0.5 s to 5 s (1 s)

*The ranges represent the normal spectrum used for mapping and were varied as necessary during mapping the mini-grid. Pulse width, current and frequency were varied systematically in the first part of the trial and subjects reported changes in sensation. Values in brackets are starting point values*.

**Table 3:** Hand receptive fields and selected stimulation site.

Subject ID	Non-overlapping locations (%)*	Unique discriminable	Receptive fields	Area of hand stimulated	Description			
01	25.0	25.0	All fingers	Ventral and dorsal surface of tips of digits 2–3	“Tinging”, “tickling”
02	27.3	27.3	Digits 2, 3 and 4, and palm	Ventral surface of digits 1–2	“Buzzing”
03	33.3	83.3	All fingers and palm	Ventral and dorsal surface of tips of digits 3–5	“Electricity”
04	16.7	66.7	All fingers and palm	Ventral surface of digits 2–5	“Soft”, “trembling”, “like it’s moving”
05	100.0	100.0	Digit 5	Lateral/proximal surface of digit 5 and palm	“Itching”, “tickling”, “pulsing”
06	46.2	76.9	All fingers	Ventral surface of tip of digits 1–2	“Shock”
07	37.5	100	Digits 1, 2, 4 and 5, and palm	Ventral surface of tip of digit 2	“Electricity”
08	41.7	58.3	All fingers	Ventral surface of tip of digit 2	“Light tapping”
09	50.0	100.0	Digits 2, 3 and 4, and palm	Center of palm	“Tingling”

Percentage of non-overlapping receptive fields representing those fields that were not repetitive; percentage of unique discriminable receptive fields from sensory responses on hand (i.e., those responses that may have been partly repetitive, but contained unique areas), and all areas of the hand where a receptive field was identified. The area of hand stimulated refers to the location where subjects consistently reported sensation for the selected stimulation site. Descriptions indicate the subjects’ own verbal explanation of the percepts elicited by the area of hand stimulated. *Excluding whole hand responses.

### Stimulation Parameters Mapping

Once the pair of contacts were chosen, the parameters were varied systematically. We varied pulse width, frequency and current amplitude using stimulation parameters described in Table 2. As one parameter was tested, all other parameters were held constant at values that showed reliable stimulation during the grid-mapping session (determined by the epileptologist, an example set of parameters would be: stimulation duration of 1 s, frequency of 50 Hz, amplitude of 3 mA, and variations in pulse-width as outlined in Supplementary Table S5). The subjects verbally reported whether they felt the stimulation, described the elicited sensation, and reported any changes in this sensation as parameters were varied. The parameter mapping was stopped if subjects reported any discomfort or pain, or if involuntary movements, twitches, or contractions were elicited with the stimulation.

### Behavioral Tasks With Cortical Stimulation

Subjects performed two sensory feedback-driven target-acquisition tasks. During these tasks, the subjects were asked to move their hand over two-by-one or three-by-three grids, searching for grid locations (“targets”) identified by electrical stimulation (Figures 2B,C). The same stimulation parameters were used across all trials. In the first paradigm, subjects discriminated between left and right targets in a two-by-one grid (Figure 2B). In the second paradigm, a target orientation discrimination task, the subjects explored a three-by-three grid (Figure 2C) to find three hidden targets arranged in a line. Subjects verbally reported when each target was identified, and indicated the orientation of the line defined by the three targets. For example, Figure 2C shows a “diagonal” underlying direction, where the subject would receive stimulation when moving over each of the green circles (circles were always blue for the patients). Each subject performing this task completed between 25 and 50 trials of each paradigm, and trials in both tasks were self-initiated and self-paced.

## Results

No adverse events, including seizures or significant discomfort, occurred during any of the tests. Occasionally, patients reported having an uncomfortable or strange sensation, but indicated no sense of pain. The sessions were terminated early for two subjects. Subject 04 (S04) reported feeling fatigued after completing the first behavioral task, and S09 reported a non-painful heat sensation after receptive field mapping and stimulus parameter testing.

### Somatotopy of Receptive Fields

**Figure 3:**
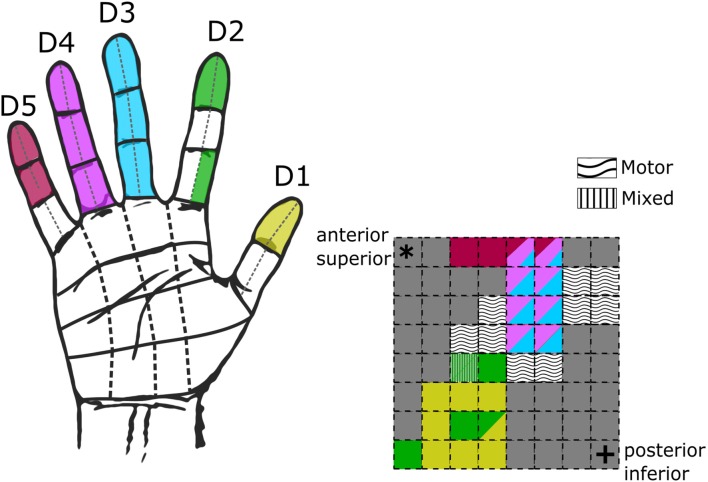
Hand receptive fields from grid mapping in a single subject. Schematic of hand coverage from initial grid mapping with subject S08 at 50 Hz, 500 μs, 2–4 mA. On the left, a hand diagram with the overlaid color-coded receptive fields from all tested electrodes. On the right, a schematic of the mini-ECoG grid following the same color code displayed on the hand to display in which digits the subject reported the sensations. Multiple colors in a single electrode indicate sensations across multiple digits. Wavy and stripped textures illustrate electrodes which elicited motor-only and mixed (motor-sensory) responses. Corner asterisk and cross markers on the grid indicate the anatomical orientation of the implanted grid.

**Figure 4:**
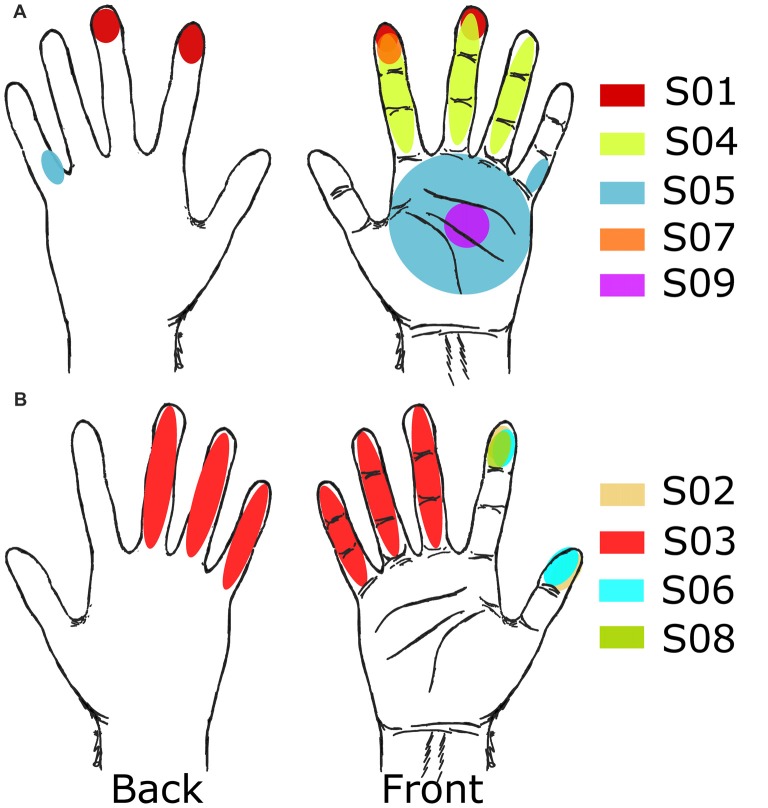
Receptive fields from grid mapping. Selected receptive fields after grid mapping for all subjects. **(A)** Subjects with right side implants. **(B)** Subjects with left side implants.

We identified sensory receptive fields on the hand, palm and fingers during the initial grid mapping (Table 3). Figure 3 shows an example of the receptive fields for a single subject (S08), and Figure 4 shows the selected receptive fields of the entire population overlaid. Some electrode pairs induced sensations in single digits, and others had receptive fields that spanned across multiple neighboring digits. Four subjects had receptive fields covering all five digits simultaneously (this was universally described as a vague sensation across the entire hand). Table 3 includes a summary, for each subject, of the number of nonoverlapping locations and the unique discriminable receptive fields (i.e., partially duplicated areas that contain unique somatotopic fields; for instance, if one electrode pair covered digit 2 and another included digit 2 and 3, this was a unique discriminable receptive field, but was not a nonoverlapping location).

For the electrodes that elicited a somatosensory percept, Table 3 also provides a description of the receptive fields covered. Although most receptive fields were overlapping, subjects could still use differences between fields to discriminate between them, for example, between sensation on ventral tip of digit 2, and sensation spanning the ventral tips of digits 2 and 3. Of the somatosensory electrodes, 17% induced sensations in the palm or whole hand, and 68% induced receptive fields exclusively on the digits. Moreover, for most subjects, as stimulation moved laterally and anteriorly on the mini-ECoG grid, the receptive fields moved from pinky to thumb, consistent with other studies with ICMS in NHPs and humans (Kaas et al., 1979; Flesher et al., 2016; see Figure 3).

Electrode pairs with well-defined regions on the tip of the fingers and lateral, ventral, or dorsal surfaces of single or multiple neighboring digits (focusing on the index finger and thumb) were favored for further testing (Table 3, Figure 4). Only one subject, S09, did not have a stable representation of digits available but did report consistent sensations on the palm (Figure 4A).

### Effects of Stimulation Parameters

Parameter values were systematically increased within the ranges specified in Table 2, and all stimulations were 1 s in duration. Subjects reported a variety of sensations but the most common descriptions were “pulsing”, “electricity”, and a “feeling of movement” with no visible movement (Supplementary Tables S5–S7). Stimuli were delivered sequentially with increasing pulse width, amplitude, or frequency. Responses were classified by comparing them to the immediate previous response, and comparisons were separated into four categories: (1) *first time*, when subjects had not reported any percept with the previous parameter settings; (2) *stronger*, when subjects reported the same sensation and receptive field but with more intensity; (3) *different*, when subjects reported a different sensation from that elicited with the previous parameter settings or a jump in location (size increase within the same finger or region was not taken as a different receptive field); and (4) *same*, when subjects reported the same location, sensation and intensity.

**Figure 5:**
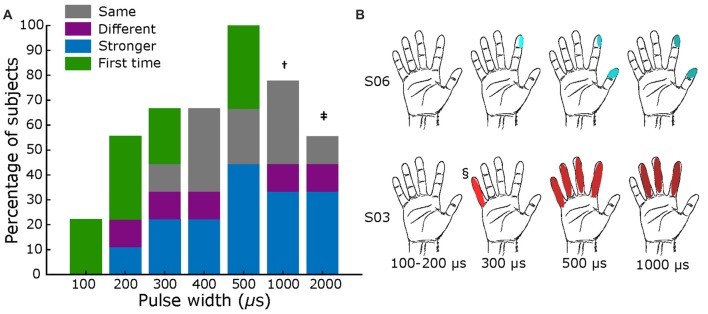
Pulse width mapping. **(A)** Percentage of subjects that reported sensations across tested pulse widths. Blue section shows fraction of reports of stronger sensation, purple section represents fraction of different sensations or different sensory field, green sections show if it was the first time feeling a percept, and gray section shows those were sensation was the same. All comparisons are with previous lower value. Total subjects: nine, fixed current amplitude = 2–3 mA, and fixed frequency = 50 Hz († value tested in six subjects; ‡ tested in five subjects). **(B)** Exemplar receptive fields for S03 and S06, darker shades represent a stronger sensation. Main elicited sensations were “pressure” for S06, and “electricity” for S03. § Sensation different from main: feeling of movement.

During pulse width variation, a minimum width of 200 μs was necessary for most subjects (56%) to report any sensation, as shown in Figure 5A. At 500 μs, all subjects reported having some type of sensation. Most subjects felt an increase in intensity as the pulse width increased (Supplementary Table S5), as displayed in Figure 5B as darker shades in the filled receptive fields. However, some subjects also reported changes in the type of sensation (e.g., S03 transitioned from “movement” to “electricity”), and variations in the receptive field location. As shown in Figure 5B for S03 and S06, the receptive fields extended to neighboring digits, as pulse width increased.

**Figure 6:**
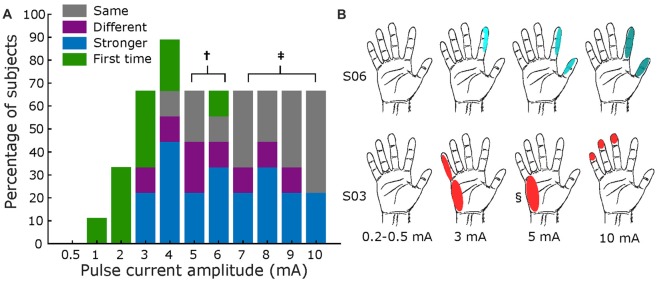
Amplitude mapping. **(A)** Percentage of subjects that reported sensations across tested current amplitudes. Same format as Figure 5. Total subjects: nine, fixed pulse width = 500 μs, and fixed frequency = 50 Hz († values tested in seven subjects; ‡ tested in six subjects). **(B)** Exemplar receptive fields for S03 and S06, darker shades represent a stronger sensation. Main elicited sensations were “shock” for S06, and “feeling of movement” for S03. § Sensation different from main: pulsing.

Figure 6 summarizes results for current amplitude testing. Panel A displays the results across all subjects, where a current of 3 mA marked the threshold at which most participants (67%) reported a sensation. As with pulse width testing, subjects generally reported feeling an increase in intensity of the same sensation as the amplitude increased, with some variations in receptive field size. Figure 6B shows exemplar receptive field and perceived intensities for S03 and S06, where darker shades illustrate stronger reported sensations. Most subjects’ responses resembled S06, but some had varying field size as amplitude increased, as shown for S03.

**Figure 7:**
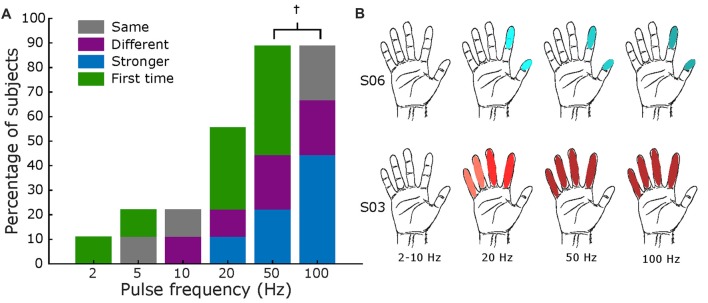
Frequency mapping. **(A)** Percentage of subjects that reported sensations across all tested frequencies. Same format as Figures 5, 6. Total subjects: nine, fixed amplitude 2–3 mA, and fixed pulse width = 500 μs († values only tested in eight subjects). **(B)** Exemplar receptive fields for S03 and S06, darker shades represent a stronger sensation. Main elicited sensations were “pressure” for S06 and “electricity” for S03.

Similarly, Figure 7 displays results for frequency mapping. A minimum of 20 Hz was necessary for most subjects (56%) to report a sensation, as shown in Figure 7A. Increasing frequencies elicited, for most subjects, stronger intensities of the same sensation, as shown in Figure 7B. One of nine patients specifically noted the increase in frequency was “faster” (S08, Supplementary Table S7), while two subjects asserted that the percepts from 50 Hz to 100 Hz were the same.

### Target Acquisition Tasks

In the target acquisition tasks, the parameters were set to those deemed by the epileptologist to be the most robust after stimulus parameter testing. These values were typically alternating polarity, pulse width of 500 μs, frequency of 50 Hz, and amplitude of 2–6 mA, and 1-s stimulation duration.

Table 4 summarizes the results for the left/right and target-direction discrimination tasks. All subjects had 100% accuracy in the left/right discrimination task, and average success rate was 98.3% (s.d. 3.1%) in the target orientation task. Two subjects did not attempt the target orientation task. One subject did not perform any of the behavioral tasks due to a persistent heat sensation in the hand between testing trials (the feeling was not noted to be painful, but prevented further testing).

**Table 4:** Target localization and discrimination tasks results.

Subject ID	Left/Right task (correct trials/total trials)	Target orientation discrimination (correct trials/total trials)	Comments
01	50/50	25/25	
02	50/50	25/25	
03	50/50	40/40	
04	19/19	Did not attempt	Limited by subject fatigue
05	50/50	23/25	Two missed trials: subject did not feel stimulation over third target.
06	50/50	48/50	Two missed trials: subject was moving too quickly across dots
07	50/50	50/50	
08	25/25	25/25	
09	Did not attempt	Did not attempt	Subject had a persistent sensation of “heat” and “tightness” in the palm of her hand and testing could not be completed

*One hundred percent accuracy was noted on the Left/Right discrimination task and 98.3% accuracy was noted on the Direction discrimination task*.

## Discussion

In this study, electrical stimulation of somatosensory cortex through mini-ECoG grids produced artificial percepts consistently and safely. The feasibility of generating sensory perception in patients with loss of limb, spinal cord injury, stroke, or other causes has important implications for functional restoration where sensory pathways have been damaged. Although the complete restoration of function is the ultimate goal, even crude sensory inputs can have a significant impact on a patient’s functional status (Flesher et al., 2016). For example, artificial somatosensation may augment motor control of a robotic limb by providing force regulation, shape discrimination and temperature detection.

We used clinical stimulation parameters to generate the percepts of somatosensation by stimulating mini-ECoG grids placed subdurally over the hand area of S1. Sections of the grid with motor-only responses to stimulation defined the boundary between motor and somatosensory cortices. Replicable somatotopic representations of the hand were identified across most subjects (Figure 3). As summarized in Table 3, receptive fields often covered distinct sections of single or neighboring digits (e.g., distal and medial phalanges, ventral surface of tip, etc.). Only one previous study with a different high-density ECoG grid was able to get a rough somatotopic representation of the hand and arm, but could not distinguish receptive fields within the hand (Hiremath et al., 2017). In this study, we induced sensations in smaller, more distinct receptive fields on the hand as we stimulated different electrode pairs.

The qualitative character of some of our reported sensations was similar to those found in previous studies with both standard (Johnson et al., 2013) and high-density ECoG grids (Hiremath et al., 2017). However, some of our subjects also described the elicited sensations with descriptors similar to those reported with microelectrode stimulation, for example, as a “light tap” or a sensation of “pressure” (Flesher et al., 2016). Peripheral nerve stimulation has also shown more natural sensations, including percepts such as “pressure”, “natural tapping”, “light moving touch” and “vibration” (Tan et al., 2014; Delhaye et al., 2016; Graczyk et al., 2016), although this modality would not be available to spinal-cord injury patients. However, the patterns used to generate these percepts through peripheral nerve stimulation might serve to inform and enrich cortical stimulation protocols. Further systematic testing is required to understand how percept qualities depend on the subject and the stimulation modality, and these issues are the topics of ongoing investigation.

As the parameters of pulse width, current amplitude, and frequency were increased, there was an associated increase in the reported intensity of the percepts (Figures 5–7 and Supplementary Tables S5–S7). This finding is in accordance with other human cortical stimulation paradigms (Johnson et al., 2013; Cronin et al., 2016; Flesher et al., 2016), and comparable to reports from NHPs of increased detection probability as stimulation parameters increased (Kim et al., 2015a,b). In contrast to Hiremath et al. (2017), we found that an increase in pulse width duration was most likely to produce an increase in the strength of the perceived sensation (accounting for 44% of responses) than a change in the type of percept or receptive field (15% of responses). Similarly, higher current amplitudes evoked a stronger percept more often (43% of responses) when compared to a previous stimulation. Although percepts were more variable with lower stimulation frequencies (<20 Hz), we found a direct relationship (*r* = 0.998, *p* = 0.029. Pearson correlation coefficient) between stimulation frequency and perceived intensity from 20 Hz to 100 Hz (43% of total responses) for most subjects (two of eight subjects reported no change between 50 Hz and 100 Hz). Prior work in NHPs found reliable percepts with stimulation frequency as low as 10 Hz (Romo et al., 2000), perhaps due to differences in the stimulation modality (microelectrodes vs. mini-ECoG) or experimental protocol.

Our results show that multiple subjects can reliably detect and discriminate artificially evoked sensations. In addition to achieving nearly 100% accuracy in both behavioral paradigms (Table 4), most subjects provided near-immediate responses to the stimulation-induced percepts. These results demonstrate performance similar to prior studies in both human and NHP (Romo et al., 1998, 2000; Cronin et al., 2016). The speed and accuracy with which the subjects identified percept locations could enable rapid online correction while operating a robotic prosthesis. Fast, accurate touch discrimination of distinct hand locations would allow finer motor control and shape discrimination.

To our knowledge, this is the first study of cortical stimulation in humans through mini-ECoG electrodes that has systematically examined the effect of stimulation parameters on sensation across multiple patients. Recent work with intracortical arrays has shown detection thresholds and qualitative assessment of the stimulation while varying current amplitude (Flesher et al., 2016), but not with pulse width or frequency. A similar study with an ECoG grid tested the effects of different parameters but was limited to one subject, and did not explore the use of these percepts in a behavioral paradigm (Hiremath et al., 2017). Here, we obtained reproducible hand representations across patients with the mini-ECoG grid, to broadly estimate parameter thresholds across multiple subjects, as shown in Figures 5–7, and to assess qualitatively how percepts varied with stimulation parameters (Supplementary Tables S5–S7).

One of the limitations of this study is the manual process for delivery of stimulation. In all four missed trials (occurring across two subjects), performance may have been constrained by either the epileptologist’s ability to react to the subject’s movement speed, or errant target identification in the manual stimulation command produced by the epileptologist. However, the accuracy and consistency of the subjects’ responses suggest that the manual stimulation did not significantly affect their ability to detect and discriminate the sensory percepts.

Subjects participating in this study suffer from epilepsy, a pathology that could potentially alter cortical networks responsible for somatosensation. While this potential must be acknowledged, the results of this article illustrate that the subjects could use the percepts corresponding to cortical stimulation to discriminate both locations and levels of intensity.

While the clinical indication for surgical monitoring of epilepsy patients serves as the foundation for conducting this study, aspects of the clinical environment constrained the experimental paradigm. The ECoG stimulation parameters used in this study were limited to the standard clinical mapping parameters. These parameter ranges are intentionally conservative to establish margins of safety. In addition, because subjects were tested in the ICU during clinical monitoring, experiments were limited by both patient availability and their treatment schedules. Despite these challenges, the data described in this study clearly demonstrate that ECoG based cortical stimulation can be delivered safely, and that subjects are able to accurately utilize these artificially generated percepts to perform behavioral tasks.

Evoked sensations described in this study are simple, artificial and convey limited information, and yet location and intensity represent fundamental aspects of somesthetic input. Additional work will be required to examine whether these types of sensations can be used to improve robotic limb control, but this study represents an encouraging step towards the goal of bidirectional BMIs.

## Author Contributions

BL, CL and RA conceived the original idea and experiments. BL, DK, MAS, SK, DB, TD, CK and CH planned and operationalized the experiments. BL, DK and MAS carried out the experiments. BL, DK, MAS, SK and CL contributed to the interpretation of the results. BL, DK, MAS, SK and RA took the lead in writing the manuscript. All authors provided critical feedback and helped shape the research, analysis and manuscript.

## Conflict of Interest Statement

The authors declare that the research was conducted in the absence of any commercial or financial relationships that could be construed as a potential conflict of interest.
