# Supplementary material for: Engineering Artificial Somatosensation Through Cortical Stimulation in Humans
Source: Front Syst Neurosci. 2018 Jun 4;12:24. doi: 10.3389/fnsys.2018.00024 (PMC5994581; doi:10.3389/fnsys.2018.00024)
Supplement: Supplementary file 1 [file Table_5.DOCX]

**Supplementary material - Table 5**

| **Pulse width** | **S01** | **S02** | **S03** | **S04** | **S05** | **S06** | **S07** | **S08** | **S09** |
| --- | --- | --- | --- | --- | --- | --- | --- | --- | --- |
| **100 μs** | No sensation | No sensation | No sensation | No sensation | Pulse in the abdomen on left | No sensation | No sensation | No sensation | “Tingling” on center of palm |
| **200 μs** | Faint “buzz” on digits 2-3 | No sensation | “Moving” on digit 5, no movement observed | No sensation | Pulse in the gluteal region on left | No sensation | Light feeling of “ants crawling” on ventral tip of digit 2 | No sensation | Slightly stronger “tingling” on center of palm |
| **300 μs** | Stronger buzzing | Slight tingling in thumb | “Electricity” on digits 2-5 | No sensation | No sensation | “pressure” “squeezing on side of digit 2 only | “Electricity” on ventral tip of digit 2 | No sensation | Stronger of the same feeling |
| **400 μs** | Greater sensation | Slight tingling in index finger | “Electricity” on digits 2-4 | No sensation | No sensation | Stronger “pressure” on side of digit 1-2 | “Electricity” on ventral tip of digit 2 | No sensation | No change, “tingling” on center of palm |
| **500 μs** | Stronger tingling | Stronger tingling in thumb and index finger | “Electricity” on digits 2-5, but stronger | “Soft”, “trembling” | “Pulse” on dorsum of hand and elbow | Similar sensation | Stronger “electricity” on ventral tip of digit 2 | “Tapping” on ventral surface of tip of digit 2 | No change, |
| **1000 μs** | Stronger sensation | Index finger moving involuntarily | “Electricity” on digits 2-4 | “Movement”, no movement observed | “Pulse” on lateral region of digit 5 and hypothenar eminence | Stronger “pressure” | Movement | Stronger “tapping” sensation on ventral surface of tip of digit 2 | “Tingling” spread to pam and ventral surface of digits 1-5 |
| **2000 μs** | Stronger tingling | Whole hand contraction | N/A | “Moving”, no movement observed | “Pulse” on elbow, web of thumb and digit 1 | Stronger “pressure” | N/A | Stronger “tapping” sensation | N/A |

Supplementary Table 5. Pulse width variations. Summary of reported sensations as the pulse width was changed. When varying pulse width, the other parameters were constant: (polarity: alternating, current: 2 mA, rate: 50 Hz). *Quotes indicate descriptions from the subjects.
