# Supplementary material for: Engineering Artificial Somatosensation Through Cortical Stimulation in Humans
Source: Front Syst Neurosci. 2018 Jun 4;12:24. doi: 10.3389/fnsys.2018.00024 (PMC5994581; doi:10.3389/fnsys.2018.00024)
Supplement: Supplementary file 2 [file Table_6.DOCX]

**Supplementary material - Table 6**

| Current amplitude | S01 | S02 | S03 | S04 | S05 | S06 | S07 | S08 | S09 |
| --- | --- | --- | --- | --- | --- | --- | --- | --- | --- |
| 0.2-0.5 mA | No sensation | No sensation | No sensation. | No sensation | No sensation | No sensation | No sensation | No sensation | No sensation |
| 1 mA | No sensation | No sensation | “Pulsing” on left leg, then moved to right leg | No sensation | No sensation | No sensation | No sensation | No sensation | No sensation |
| 2 mA | Mild tingling on fingers | Pinky tingling | No sensation. | No sensation | No sensation | No sensation | “electricity” on ventral tip of digit 2 | No sensation | No sensation |
| 3 mA | More tingling on palm | Thumb pulsating | “Movement” on digit 5 and hypothenar eminence, no movement observed. | No sensation | “Pulsing” and “movement” without observable movement. | Very light “shock” on digit 2 side and tip | Stronger “electricity” on ventral tip of digit 2 | No sensation | No sensation |
| 4 mA | More intense buzzing | 3-4-5 finger tingling | “Movement” and “pulsing” on digit 5. | No sensation. | Stronger “pulsing” and “movement”. | Stronger “shock” further down side of digit 2 | Stronger “electricity” on ventral tip of digit 2 | “Tapping” on ventral surface of tip of digit 2 | “Tingling” on center of palm |
| 5 mA | Actual finger contraction | Whole hand pulsing | “Pulsing” on hypothenar eminence. | No sensation. | Stronger “pulsing” and “movement”. | “Shock” on digit 1-2, side of finger | Movement | Stronger “tapping” sensation on ventral surface of tip of digit 2 | No, change, “tingling” on center of palm |
| 6 mA | N/A | Actual finger contraction | “Knuckle pressure” on digits 3-4. | “Moving” throughout hand, no movement observed | Stronger “pulsing” and “movement”. | “shock” on digit 1-2, side of finger | N/A | Stronger “tapping” sensation | Faster “tingling” on center of palm |
| 7 mA | N/A | N/A | “Jumping” sensation on palm. | “Knuckle pressure” on digits 3-5. | Stronger “pulsing” and “movement”. | “shock” down further on digit 2 | N/A | Stronger “tapping” sensation | No change |
| 8 mA | N/A | N/A | “Moving” on digit 5, no movement observed. | “Jumping” sensation on palm. | Stronger “pulsing” and “movement”. | Stronger “shock” on digit 1-2, tip and side of finger | N/A | Stronger “tapping” sensation | No change |
| 9 mA | N/A | N/A | “Moving” and “trembling” on digit 5. | “Moving” on digit 5, no movement observed. | Stronger “pulsing” and “movement”. | “shock” on digit 1-2 extending more to ventral surface of digit 2 | N/A | Stronger “tapping” sensation | No change |
| 10 mA | N/A | N/A | “Moving” on tips of digits 3-5. | “Moving” and “trembling” on digit 5. | Stronger “pulsing” and “movement”. No movement observed. | “shock” on digit 1-2 side and ventral surface | N/A | Stronger “tapping” sensation | No change |

Supplementary Table 6. Variation in current amplitude. Summary of reported sensations as amplitude was changed. When varying amplitude, the other parameters were constant (polarity: alternating, pulse width 500 μs, rate: 50 Hz). *Quotes indicate descriptions from subjects.
