# Supplementary material for: Engineering Artificial Somatosensation Through Cortical Stimulation in Humans
Source: Front Syst Neurosci. 2018 Jun 4;12:24. doi: 10.3389/fnsys.2018.00024 (PMC5994581; doi:10.3389/fnsys.2018.00024)
Supplement: Supplementary file 3 [file Table_7.DOCX]

**Supplementary material - Table 7**

| Frequency | S01 | S02 | S03 | S04 | S05 | S06 | S07 | S08 | S09 |
| --- | --- | --- | --- | --- | --- | --- | --- | --- | --- |
| 2-4 Hz | No sensation | No sensation | No sensation | No sensation | No sensation | No sensation | No sensation | No sensation | “Tingling” on center of palm and ventral surface of digits 1-5 |
| 5 Hz | No sensation | No sensation | No sensation | No sensation | “Pulse” on digit 1. | No sensation | No sensation | No sensation | No change |
| 10 Hz | No sensation | No sensation | No sensation | No sensation | “Movement” felt in elbow, no movement observed. | No sensation | No sensation | No sensation | No change |
| 20 Hz | No sensation | No sensation | "electricity" on digits 2, 3, 4, 5, very slight intensity | “Moving” on digits 3-5. | “Pulse” on digit 5 and hypothenar eminence. | “light pressure” on digits 1-2 | No sensation | No sensation | Stronger sensation of tingling |
| 50 Hz | Starting to tingle | Palm tingling | stronger "electricity" digit 2, 3, 4, 5 | “Felt hot” on digits 3-5. | Movement observed in digit 5. | Stronger “pressure” on digits 1-2 | “Electricity” on ventral surface digit 1 and 2 | “Tapping” on ventral surface of tip of digit 2 | Stronger sensation of tingling |
| 100 Hz | Strong tingling, hand moving involuntarily | Tingling in digits 4 and 5 | same "electricity" digit 2, 3, 4, 5 | “Felt hot” on digits 3-5, same intensity as previous. | “Movement” felt at dorsum of hand, no movement observed. | Stronger “pressure” on digits 1-2 | Movement | Stronger and faster “tapping” sensation on ventral surface of tip of digit 2 | Stronger sensation of tingling |

Supplementary Table 7. Frequency variations. Summary of reported sensations as pulse frequency were changed. When varying frequency, the other parameters were constant (polarity: alternating, pulse width 500 μs, current: 2 mA). *Quotes indicate descriptions from subjects.
